# The EU Interreg Project “GEREMIA” on waste management for the improvement of port waters: results on monitoring the health status of fish as bioindicator

**DOI:** 10.1007/s11356-023-25587-4

**Published:** 2023-01-31

**Authors:** Anna Reboa, Giovanni Besio, Laura Cutroneo, Irene Geneselli, Stefania Gorbi, Alessandro Nardi, Maria Elena Piccione, Francesco Regoli, Marco Capello

**Affiliations:** 1 DISTAV, University of Genoa, 16132 Genoa, Italy; 2 DICCA, University of Genoa, 16145 Genoa, Italy; 3 DISVA, Polytechnic University of Marche, 60131 Ancona, Italy; 4 ISPRA, 00144 Rome, Italy

**Keywords:** Monitoring, Marine waters, Fish, Bioindicators, PAHs, Trace metals, EROD activity, Fish histopathology

## Abstract

Highly anthropized areas as ports represent complex scenarios that require accurate monitoring plans aimed to address the environmental status. In this context, the activities of the EU Interreg Project “GEstione dei REflui per il MIglioramento delle Acque portuali (GEREMIA)” were focused on comparing sites differently affected by human presence, as the Port of Genoa and the natural area of the S’Ena Arrubia fishpond: a panel of analyses was carried out on Mugilidae fish sampled in these two areas, aimed to address trace metal accumulation in the liver, gills, and muscle, as well as cytochrome P450 (CYP450) induction in liver and biliary polycyclic aromatic hydrocarbon (PAH) metabolites, and histopathological alterations in the liver and gills. Chemical analyses in the liver, gills, and muscle of specimens collected in the port area showed an overall higher degree of trace metal contamination compared to the natural fishpond, and similar results were obtained in terms of CYP450 induction and biliary PAH metabolites, suggesting a higher exposure to organic compounds. In addition, histopathological analyses revealed a significant alteration and then a loss of functionality of liver and gill tissue in individuals from the port. Overall, this study describes the complex environmental pollution scenario in the Port of Genoa, confirming the importance of using multidisciplinary approaches and different types of analyses to address both the presence and the effects of contaminants in marine environments.

## Introduction

Scientific interest in monitoring the marine environment has increased in the last decades, being this considered as one of the main sinks of anthropogenic pollutants (Vikas and Dwarakish 2015). In this context, port areas are severely affected by contaminants reflecting the high levels of anthropization (Bonsignore et al. 2018; Wooldridge 1999): indeed, ports are characterized by a strong presence of commercial and industrial activities and are strictly connected with cities nearby and with the hinterland and therefore gather different types of discharging inputs, both urban and industrial, and accidental loss of pollutants and solid waste is likely to occur (d’Errico et al. 2021; Homsombat et al. 2013). Port authorities’ policies are therefore improving the aspects related to the management of environmental issues, enhancing green solutions and intensifying prevention and monitoring activities. In this context arises the EU Interreg Italy-France Maritime 2014–2020 Project “GEREMIA-GEstione dei REflui per il MIglioramento delle Acque portuali (waste management for the improvement of port waters)” (http://interreg-maritime.eu/web/geremia), which started in 2018 and ended in 2021. The GEREMIA Project gathered scientists and professionals from Italian and French partners, with the aim to develop new tools for the management of environmental emergencies related to port waters. Several monitoring actions took place during the project, including biomonitoring activities to collect data for the creation of an integrated index for the quality of port waters. The present study focuses on biomonitoring campaigns carried out in the Port of Genoa: this port is located on the Ligurian Coast, in the north-western Mediterranean Sea, and is characterized by the presence of various commercial and industrial activities, as well as being strictly connected with the urban area of the city of Genoa. In fact, the Port of Genoa has multiple terminals equipped for a variety of vessel and cargo types, such as containers, dry and liquid bulk, metals, and petroleum products. In addition, there are piers that handle passenger traffic, both through cruises and ferries. Finally, the Port of Genoa is known worldwide as an important center for ship repair of large vessels, both commercial, passenger, and pleasure craft (Cutroneo et al. 2017). The intense anthropization of the area led to the presence of several inputs of water contamination, including urban and industrial discharging points and the mouth of two water streams, namely, Polcevera and Bisagno Torrent (Cutroneo et al. 2017).

Such complex scenario needs accurately developed monitoring plans aiming to precisely assess the degree of environmental impacts: these should not only refer to the presence of contaminants in water column and sediments but also address accumulation and possible effects of such contaminants in wild organisms; in fact, the investigation of the health status of organisms is a useful parameter for assessing the level of contamination, reflecting the quality of the environment where they live and feed (Gupta and Singh 2011). Thus, integrative approaches that include biological monitoring and multidisciplinary investigations are nowadays considered an important tool for detecting critical environmental conditions (Dalzochio and Gehlen 2016) and are strongly encouraged by European Directives such as the Marine Strategy Framework Directive (European Commission 2008a) and the Water Framework Directive (European Commission 2000). Biological monitoring is performed through target organisms, defined as bioindicators, which are tolerant to the presence of contaminants, but at the same time sensitive enough to show sub-lethal changes in their physiological state (Zhou et al. 2008), which are detectable through biological investigations (biomarkers), e.g., histological and enzymatic techniques (Valavanidis and Vlachogianni 2010). Nonetheless, while biomonitoring is known as a useful tool to investigate a contaminated area, this should not focus only on a single biomarker, which may lead to an underestimation or misinterpretation of the overall environmental impact (Van der Oost et al. 2003).

In this study, a biological monitoring activity was conducted using fishes of the Mugilidae family; these are considered as good bioindicators because of their ecological role, their commercial value, and their importance for human consumption (Dalzochio and Gehlen 2016; Zhou et al. 2008). Nonetheless, fish from the Mugilidae family were selected due to their extensive presence in contaminated areas as ports, their characteristics related to living and feeding habits, and their commercial importance. Mugilidae fish commonly live in coastal environments, are able to feed both along the water column and from the sediment, and directly exploit food resource provided by primary producers, thus having a strong impact on the flow of energy and organic matter in marine environments (Almeida 2003; Carpentier et al. 2014; Pedro et al. 2008; Whitfield et al. 2012).

A panel of different typologies of analyses was conducted on collected specimens, including levels of trace metals in the gills, liver, and muscle, induction of cytochrome P450 1A biotransformation pathway (EROD activity) in liver and PAH metabolites in bile, and histopathology of livers and gills. Trace metals were analyzed since these contaminants are frequently detected at relevant concentrations in port waters and sediment (Jahan and Strezov 2018). In fact, metal contamination is also known to occur in the Port of Genoa, and several studies have been conducted previously to investigate this problem and find innovative solutions (Cecchi et al. 2019a, 2019b; Cutroneo et al. 2017; Lucchetti et al. 2017). Beside trace metals, polycyclic aromatic hydrocarbons (PAHs) and more in general organic contaminants represent a relevant issue in port systems, reaching high levels of contamination: the exposure to these compounds is known to promote cytochrome P4501A biotransformation pathway, which has been extensively used as a marker of exposure to organic contaminants measuring EROD activity (Benedetti et al. 2007; Regoli et al. 2003). The biotransformation of these compounds may lead to the formation of metabolites, as naphthalene-, pyrene- and benzo[a]pyrene-like metabolites that are often measured in bile as widely used and recognized markers of PAH exposure in fish (Baali et al. 2016; Escartín and Porte 1999). Regarding histopathology approach, it has been exploited in literature to investigate the overall alteration of the physiological status of entire organs (Au 2004), despite being a semi-quantitative and non-specific biomarker. On the whole, the multidisciplinary set of analyses proposed in the present study would represent a comprehensive survey of a port marine environment.

Investigation was conducted selecting the fishpond of S’Ena Arrubia (Sardinia-Italy), as a comparison and reference site, to better understand how the environment influences the health status of organisms: indeed, the fishpond represents a marine environment with characteristics similar to those of the port of Genoa, but with a considerably lower degree of anthropization and therefore contamination. In addition, the fishpond of S’Ena Arrubia is exploited for commercial purposes, especially regarding the fish family of Mugilidae. As a whole, obtained results will provide insights on the usefulness of biomonitoring approaches using Mugilidae fish to assess the contamination of differently anthropized marine environments.

## Material and methods

Fish specimens were collected during four campaigns, two at the Port of Genoa (Lat. 44.412°, Lon. 8.919°) and two at the fishpond of S’Ena Arrubia (Lat. 39.828°, Lon. 8.554°). Sampling activities at the Port of Genoa were held on 17 May 2019 and 7 July 2020, prior authorization of the Italian Ministry of Agricultural, Food and Forestry Policies. The same authorization also permitted the sampling activities at the fishpond of S’Ena Arrubia that took place on 3 June 2019 and 10 July 2020. Fish were collected with nets and kept alive in seawater until sacrifice, performed by percussive stunning, and completed by cervical dislocation on unconscious organisms, following European guidelines (European Commission 2010). A total number of 76 individuals were caught during the four sampling campaigns, of which 20 from the 2019 campaigns and 18 from the 2020 campaigns, both from the port of Genoa and the fishpond of S’Ena Arrubia, belonging to the following species of Mugilidae family: *Chelon ramada*, *Chelon auratus*, and *Mugil cephalus*. After weighing (total weight in g) and measuring (total and standard length in cm), organisms were dissected, and gills, liver, gallbladder, and muscle excised. The sex of individuals was visually identified when possible, according to the degree of maturity of the gonads. Storage of samples depended on the different types of analysis to be performed. Samples of gills, liver, and muscle for chemical analysis were stored at – 20 °C, while a small portion of each liver sample and the gallbladder of each fish were immediately frozen in liquid nitrogen and stored at – 80 °C for CYP450 induction (EROD activity) and PAH metabolite analyses, respectively. Histopathological analysis was carried out on gill and liver samples that were immediately fixed in Bouin Solution and acetic acid (20:1) and stored in 70% ethanol after 24 h. Fulton’s condition factor (*K*) was calculated according to Htun-Han (1978) equation with the following formula:

$$K = (W*100)/{L}^{3}$$

where *W* is weight (g) and *L* is the total length (cm).

### Chemical analyses

Chemical analyses were carried out by the laboratories of Lifeanalytics S.r.l (Italy), and the organs were investigated regarding their content of aluminum (Al), arsenic (As), cadmium (Cd), chromium (Cr), copper (Cu), iron (Fe), lead (Pb), manganese (Mn), mercury (Hg), nickel (Ni), strontium (Sb), and zinc (Zn). Trace metals were analyzed using inductively coupled plasma optical emission spectroscopy (USEPA Method 6010D, Optima 8300) of muscle, liver, and gill tissues, after prior digestion of samples with aqua regia (USEPA Method 3050B corrected with protocol UNI EN 13,657:2004 for organic samples).

### CYP450 induction and PAH metabolite analyses

Liver and gallbladder samples stored at – 80 °C were exploited for different analyses: the ethoxyresorufin-O-deethylase (EROD) assay was performed on liver samples to evaluate the enzyme activity of cytochrome P4501A isoform, while gallbladder samples were processed to analyze polycyclic aromatic hydrocarbon (PAH) metabolites in bile, such as naphthalene-like, pyrene-like, and benzo[a]pyrene-like (B[a]P) metabolites, as follows.

Ethoxyresorufin O-deethylase (EROD) activity was spectrofluorometrically assayed according to previously published methods (Gorbi et al. 2005). Individual fish livers were homogenized (1:5, *w*/*v*) in 100 mM K-phosphate buffer pH 7.5, containing 0.15 M KCl and 1 mM ethylendiaminetetraacetic acid (EDTA), and centrifuged at 12,000 × g for 15 min to obtain S9 fraction (containing both cytosolic and microsomal fraction), immediately used for determination of enzyme activity (Stagg and MacIntosh 1998). Samples were incubated at 25 °C in a final volume of 1 ml containing 100 mM K-phosphate buffer pH 7.5, 4 μM 7-ethoxyresorufin and 0.25 mM NADPH for 3 min, followed by the addition of 2 ml acetone to stop the reaction. Blank samples, prepared as described above but immediately stopped at time zero, were used as blank values to be subtracted from the sample fluorescence. Fluorometric analyses (535/585 nm) were quantified by reference to resorufin standards (0.01–1 μM), and results expressed as pmol of resorufin produced min^−1^ mg^−1^ of protein; protein content was determined according to Lowry method with bovine serum albumin (BSA) as standard (Lowry et al. 1951).

Aromatic hydrocarbon metabolites in bile samples were determined by fixed fluorescence (FF) spectrofluorometric assay (Gorbi et al. 2005). Fish gallbladders were cut, and the collected bile samples were diluted in 48% ethanol (1:1000–1:5000 to obtain linear fluorescent readings). Sample fluorescence was analyzed at excitation/emission wavelengths 290/335, 341/383, and 380/430 for naphthalene-like, pyrene-like, and B[a]P-like metabolites, respectively, and metabolites quantified using respectively 1-naphthol, 1-OH-pyrene, and B[a]P as reference standards. The absorbance of bile samples diluted in EtOH 48% (1:100) at 380 nm was used to normalize metabolites to biliverdin content (Gorbi et al. 2005; Ruddock et al. 2003).

### Histopathological analyses

Gill and liver samples, previously stored in 70% ethanol, were subjected to dehydration in an ascending series of ethanol and then embedded in paraffin (Humason 1979). Subsequently, samples were cut with microtome (Leica RM 2135) to obtain 4-μm-thick slices that were stained with hematoxylin and eosin (Humason 1979) and then observed under optical microscope (20 × magnifications—Leica microscope). Histological alterations were investigated in 9 fields (330 × 245 μm) for each sample and quantified by their reversibility and extent within the section. A numerical index of gill (Ig) and liver (Il) health status for each fish was obtained. For this purpose, a semi-quantitative scoring system was applied, where an importance factor (*w*) was assigned to each alteration (alt) based on its reversibility, and the extent of the alteration within the section was quantified in each field by a score value (*a*) (Bernet et al. 1999). Liver or gill index (*I*) was then calculated for each specimen, with the following formula:

$$I=\sum alt\;(a\bullet w)$$

Score values ranged from 0 to 6, as follows: absence (0), mild occurrence (2), moderate occurrence (4), and severe occurrence (6). The importance factor was assigned to each alteration as here described: easily reversible (1); reversible in most cases if the causes cease to exist (2); irreversible (3). For each alteration, the mean score value assigned in the 9 fields was calculated and multiplied by the corresponding importance factor; the sum of the values obtained for all the alterations considered in the tissue resulted in its index value (Ig for gills and Il for liver). Histological alterations investigated in gills (Bernet et al. 1999; Mitchell et al. 2012) and their importance factor are as follows: secondary lamellae (SL) blood vessel congestion (*w* = 1); hemorrhage (*w* = 2); aneurysms (*w* = 1); granulocyte infiltration (*w* = 2); SL epithelial hypertrophy (*w* = 1); SL epithelial hyperplasia (*w* = 2); primary lamellae (PL) epithelial hyperplasia (*w* = 2); SL shortening (*w* = 1); SL adhesion (*w* = 2); SL fusion (*w* = 2); SL epithelial lifting (*w* = 1); necrosis (*w* = 3). Histological alterations investigated in liver (Bernet et al. 1999; Richardson et al. 2010; Van Dyk et al. 2012) and their importance factor are blood vessel congestion (*w* = 1), hemorrhage (*w* = 1), melanomacrophage centers (*w* = 1), granulocyte infiltration (*w* = 2), micro and macro steatosis (*w* = 1), steatosis foci (*w* = 1), hyalinization (*w* = 1), hydropic change (*w* = 1), loss of cord structure (*w* = 1), nuclei pyknosis (*w* = 3), tissue degeneration (*w* = 3), cellular necrosis (*w* = 1), and necrosis foci (*w* = 3). The health status of the organs has been classified on the base of the resulting indices, as follows: normally or very mild altered functionality (*I* ≤ 10); slightly to moderately damaged tissue (10 < *I* ≤ 20); moderately to heavily damaged tissue (20 < *I* ≤ 30); heavily to irreparably damaged tissue (*I* > 30).

### Statistical analyses

Differences between sites and campaigns in terms of weight of fish were tested using a one-way ANOVA, while a Mann–Whitney test was applied to compare length of fish. Student’s *t*-test was performed to test differences between sites regarding condition factors of specimens. Mann–Whitney test was applied to analyze trace metal content differences between sites for each tissue. Data of EROD activity and PAH metabolites were checked for normal distribution and BoxCox transformation conducted (MASS package, RStudio) when normal distribution of data was not met. Then, a linear model (2-way ANOVA) was applied to test the effect of the factors “Site” (Port of Genoa and S’Ena Arubbia), “Campaign” (2019 and 2020), and their interactions on EROD activity and PAH metabolites. A two-way ANOVA was performed, prior Kolmogorov–Smirnov test for normal distribution, to analyze values of liver and gill indices from the histopathological investigation, comparing the two different sites and campaign for each organ. Single histological alterations in gill and liver samples were tested for presence/absence using PCA analysis, investigating differences between both sites and species. The mean values of the extent of alterations in each liver and gill sample were tested for correlation with the content of different trace metals in the corresponding tissue, using Spearman’s correlation coefficient. One-way ANOVA was applied to test differences between species within sites both for histopathological indices and EROD activity. Spearman rank correlation analysis was applied in order to investigate the relationship between EROD activity and histopathological indices of liver and gills. Available data on the sex of the individuals were used to test the difference between males and females, both in terms of histopathological indices and EROD activity. The difference due to sex regarding histopathological index values was tested by one-way ANOVA, while a Mann–Whitney test was applied to any differences regarding EROD activity. The data indicating the size of the individuals (length and weight) were compared with the biomarkers analyzed (metals in different tissues, histopathological indices, EROD, and bile metabolites) by correlation, using Pearson’s index or Kendall’s Tau, depending on whether the data were distributed normally or not. If necessary, linear regression was applied to calculate the coefficient of determination.

## Results and discussion

Morphometric characteristics of fish sampled in the Port of Genoa and in the fishpond of S’Ena Arrubia during the 2019 and 2020 campaigns are summarized in Table 1. Of the total number of individuals in which sex was identified (about half of the specimens), in the Port of Genoa, 55% were females and 45% males, while in the fishpond, 74% females and 26% males were obtained. Weight of fish from the Port of Genoa ranged from 280 to 645 g and from 331 to 936 g, in the 2019 and 2020 sampling campaigns, respectively, while weight of fish from the fishpond of S’Ena Arrubia fell in the ranges 119–317 g in the 2019 campaign and 158–963 g in the 2020 campaign; the statistic revealed a highly significant difference between sites in terms of weight (one-way ANOVA, *p* value = 4.681e − 07), where fish from the port was overall heavier than from the fishpond. No significant differences were found comparing campaigns. Furthermore, individuals caught in the port area were bigger than the ones collected at the fishpond (Table 1) and total length was found to be significantly different between sites (Mann–Whitney, *p* value = 1.754e − 06) but not comparing campaigns. The difference in size may be due to the exploitation of the fishpond for commercial purposes, which limits fish growth, while fishing activities are prohibited in port areas. Fulton’s condition factor (*K*) was in the range of 0.6–1.1 for fish from both sites, and the *t*-test showed no significant difference between sites (*p* value: 0.2237). Most specimens from both the port and the fishpond had a *K* less than 1, where the condition factor is considered to indicate a good general health status when greater than 1 (Narayan Datta et al. 2013). In addition, *K* values were lower than those reported in other studies in the literature, which always reported values above 1 (Guisse et al. 2021; Jin et al. 2015; Solomon Ogunola and Ahmed Onada 2017). Therefore, the overall condition of fish was not optimal either at the site subjected to pollution or at the less anthropized site. There are several types of stresses that may have affected the health status of the individuals in the fishpond, including the fishing activity itself, which may affect the growth ability of fish. In addition, it is possible that pollutants may also be present in this environment, even if in smaller quantities than in a port environment.

**Table 1:** Morphometric characteristics (weight and total length) and results from histopathological (liver and gill indices) and molecular analyses (EROD activity and bile metabolites) on fish from the Port of Genoa (GE) and the fishpond of S’Ena Arrubia (OR)

	Site	Campaign	Min. value	Max. value	Mean	St. dev
Weight (g)	GE	May 2019	280	645	489***	112
	GE	July 2020	331	936	518***	142
	OR	June 2019	119	317	197	52
	OR	July 2020	158	963	433	284
Total length (cm)	GE	May 2019	33.5	43.0	38.5***	2.7
	GE	July 2020	34.0	44.5	38.6***	2.6
	OR	June 2019	25.0	37.5	29.4	2.9
	OR	July 2020	26.5	46.5	34.3	7.2
Liver index	GE	May 2019	4.2	20.4	12.4***	4.0
	GE	July 2020	6.2	32.4	17.7***	7.3
	OR	June 2019	0.4	13.3	5.4	2.9
	OR	July 2020	0.4	13.8	8.3	3.1
Gill index	GE	May 2019	1.6	27.8	8.7***	7.0
	GE	July 2020	1.8	21.3	8.9***	5.7
	OR	June 2019	0.7	10.2	3.7	2.6
	OR	July 2020	0.0	4.0	2.2	1.2
EROD activity (pmol min^−1^ mg prt^−1^)	GE	May 2019	74.51	387.75	193.47***/^**+++**^	80.88
	GE	July 2020	43.32	179.62	119.84***	35.28
	OR	June 2019	1.04	21.31	7.18	5.49
	OR	July 2020	5.43	29.62	14.38	7.21
Naphthalene-like metabolites (FFunits/ABS_380nm_)	GE	May 2019	117.35	753.25	336.68***	190.81
	GE	July 2020	27.29	810.46	342.41***	242.47
	OR	June 2019	31.08	138.10	89.82	27.90
	OR	July 2020	46.26	246.18	125.95	49.08
Pyrene-like metabolites (FFunits/ABS_380nm_)	GE	May 2019	86.27	3344.16	1045.54***	1067.92
	GE	July 2020	122.49	1861.11	755.55*	722.59
	OR	June 2019	96.64	353.33	203.94	73.70
	OR	July 2020	13.27	603.41	247.90	131.79
B[a]P-like metabolites (FFunits/ABS_380nm_)	GE	May 2019	57.08	383.90	173.31	117.61
	GE	July 2020	39.55	246.91	116.06	78.94
	OR	June 2019	38.58	215.56	113.93	48.30
	OR	July 2020	5.10	327.11	151.90	87.80

Asterisks and crosses highlight statistically significant differences between sites: *p value < 0.05; **p value < 0.005; ***p value < 0.001

### Chemical analyses

Results from chemical analysis showed that the common accumulation pattern was liver > gills > muscle for most of trace metals; in fact, liver is known as the organ which mostly accumulates trace metals due to its metabolic function, while muscle is the organ that less interacts with exogenous contaminants (Kalay and Canli 1999; Rajeshkumar et al. 2015). Some metal represented an exception: As and Hg content was higher in muscle than in gills, and Mn and Pb were more present in gills than in liver. Trace metals that were highly present in tissues were Fe, Mn, Zn, and Cu which are essential for fish metabolism at low levels but can be toxic at high concentrations (Yacoub and Abdel Satar 2003). Comparison of metal content between sites for each tissue was performed applying Mann–Whitney test. Iron mean values were higher in the port than in the fishpond, for liver (*p* value: 6.133e − 07), gills (*p* value: 0.01441), and muscle (*p* value: 6.595e − 09), and peaked in liver (Table 2); this might be expected since liver is a strongly vascularized tissue (Rajeshkumar et al. 2015), even if, in the present analyses, liver samples generally showed concentrations higher than several other studies where Fe content usually did not exceed 500 mg/kg, even in polluted environments (Arockia Vasanthi et al. 2013; Dural et al. 2006; Gad El-Hak et al. 2021; Jelodar, et al. 2011; Salem and Ayadi 2016; Usero et al. 2003). Concentrations on Mn were on average lower in samples from the port than from the fishpond, for both liver (*p* value: 6.595e − 09) and gills (*p* value: 0.04135) but were higher for muscle (*p* value: 7.977e − 06) (Table 2). Manganese is reported to be a constituent for bones (Yacoub and Abdel Satar 2003), and this could explain the higher content in gills that in the other tissues found in the present study. Copper resulted to reach much higher levels in liver than in gills and muscle, being an essential component of enzymes and thus crucial to metabolic functions of liver (Salem and Ayadi 2016), and its mean concentrations were significantly higher, according to the Mann–Whitney test, in samples from the port than from the fishpond in liver (*p* value: 5.639e − 05), gills (*p* value: 3.266e − 08), and muscle (*p* value: 0.01891) (Table 2). The Cu content in the liver of the sampled specimens was found to be very high compared to other studies, which found Cu concentrations below 10 mg/kg in polluted environments (Arockia Vasanthi et al. 2013; Rajeshkumar et al. 2015); however, data are widely diverse in literature, where Cu content varies in the range of 80–1040 mg/kg, and thus more in line with the results of this study (Durrieu et al. 2005; Engin 2015; Yacoub and Abdel Satar 2003; Kalay and Canli 1999; Usero et al. 2003). Regarding the Cu content in gills and muscle, however, the values obtained from this study were often below those found in the literature, which report a range of 4–9 mg/kg for gills and 1–4.5 mg/kg for muscle (Arockia Vasanthi et al. 2013; Frías-Espericueta et. 2011; Gad El-Hak et al. 2021; Jelodar et al. 2011; Laxmi Priya et al. 2011).

**Table 2:** Mean values and standard deviations (s.d.) of metal content (mg/Kg) in the liver, gill, and muscle of fish from the Port of Genoa and the fishpond of S’Ena Arrubia. Mean values and s.d. were calculated on the total of 38 fish sampled for each site in the two sampling campaigns

	Tissue	Port of Genoa		Fishpond of S’Ena Arrubia	
		Mean	s.d	Mean	s.d
Al	Liver	56.285***	61.697	16.816	15.510
	Gills	29.619***	17.853	17.716	8.246
	Muscle	17.528***	5.760	11.719	4.848
As	Liver	1.977	1.253	2.967***	1.451
	Gills	0.282	0.156	0.569***	0.587
	Muscle	0.904	0.813	0.670	0.239
Cd	Liver	0.172	0.177	0.164	0.120
	Gills	0.011	0.018	0.011**	0.004
	Muscle	0.007*	0.002	0.006	0.003
Cr	Liver	1.386***	2.959	0.434	0.945
	Gills	0.162**	0.149	0.040	0.016
	Muscle	0.060	0.039	0.047	0.024
Fe	Liver	1466.093***	933.402	595.975	589.188
	Gills	87.091*	36.779	67.577	37.988
	Muscle	16.439***	30.253	4.219	1.983
Mn	Liver	2.915	1.471	3.248***	1.318
	Gills	11.233	9.118	17.851*	14.911
	Muscle	2.693***	2.402	1.651	0.650
Hg	Liver	0.073	0.069	0.041	0.035
	Gills	0.006*	0.005	0.004	0.002
	Muscle	0.012	0.009	0.008	0.004
Ni	Liver	1.371***	1.470	0.497	0.629
	Gills	0.131***	0.098	0.073	0.041
	Muscle	0.030	0.017	0.047**	0.052
Pb	Liver	1.245***	1.281	0.117	0.181
	Gills	5.028***	3.212	0.258	0.175
	Muscle	0.131***	0.160	0.048	0.069
Cu	Liver	313.510***	277.316	114.147	58.590
	Gills	1.387***	0.726	0.770	0.217
	Muscle	0.512*	0.135	0.447	0.174
Zn	Liver	46.054	21.467	60.699*	31.993
	Gills	22.601***	5.350	17.577	2.907
	Muscle	4.398	1.046	4.982	2.797

Asterisks highlight statistically significant differences between sites: *p value < 0.05; **p value < 0.005; ***p value < 0.001

Concentration of Zn followed the common pattern that shows liver as the tissue that mostly accumulates trace metals, followed by gills and then muscle. On one hand, there was no difference in Zn mean levels in muscle between the port and the fishpond (*p* value: 0.2681) and a slightly significant difference concerning liver (*p* value: 0.01837); on the other hand, Zn mean concentrations were significantly higher in gill samples from the port than from the fishpond (*p* value: 6.674e − 06) (Table 2), although well below values reported by other studies in which gill Zn content was above 60 mg/kg (Frías-Espericueta et. 2011; Jelodar et al. 2011; Kalay and Canli 1999; Salem and Ayadi 2016; Uysal et al. 2008). Another analyzed essential metal was Cr, which is necessary for metabolism of carbohydrates (Authman et al. 2015). Chromium mean concentrations were similar in muscle from the two sites, but they were higher in the port area compared to the fishpond, for gills (*p* value: 5.75e − 11) and especially for liver (*p* value: 3.49e − 05) (Table 2). The highest concentrations of Cr found in liver samples from the Port of Genoa (max 6.1 mg/kg) were consistent with other studies carried out in polluted environments where values were in the range 1.2–11 mg/kg (Kalay and Canli 1999; Laxmi Priya et al. 2011; Norouzi et al. 2017). Toxic effects on liver includes hematological and histological alterations (Authman et al. 2015), and in this study, a positive correlation was found in liver samples, using Spearman’s coefficient, between Cr levels and granulocyte infiltration (*r*: 0.58), loss of cord structure (*r*: 0.53) and pyknosis (*r*: 0.56). Besides essential metals, high levels of Al were found, with average concentrations higher in the port compared to the fishpond for liver (*p* value: 1.599e − 07), muscle (*p* value: 1.293e − 05), and especially for gills (*p* value: 9.623e − 05) (Table 2), according to Mann–Whitney testing. Despite Al is not an essential metal, its presence in organisms is expected, since it is one of the most abundant elements on earth (Authman et al. 2015); even if high concentrations of this metal are proved to negatively affect metabolism (Sivakumar et al. 2012), no significant correlation was found in this study between Al content and alterations occurrence in fish tissues. Another essential metal which may be toxic at high concentrations is Ni (Salem and Ayadi 2016), and in the present study, its average content was higher in samples from the port than from the fishpond for liver (*p* value: 4.802e − 06) and gills (*p* value: 0.00070), but slightly higher in S’Ena Arrubia than in Genoa for muscle (*p* value: 0.0035) (Table 2). Despite concentrations of Ni found in liver were similar to literature, which report a mean concentration of 1 mg/kg (Jelodar et al. 2011; Usero et al. 2003), a significant correlation using Spearman’s coefficient was shown in liver samples between Ni levels and granulocyte infiltration (*r*: 0.52) and pyknosis (*r*: 0.59).

Regarding non-essential metals, results on Cd and Hg did not show highly significat difference between samples collected in the port and in the fishpond. Cadmium concentrations reached maximum values of 0.94 mg/kg, 0.12 mg/kg, and 0.015 mg/kg, in liver samples, gill samples, and muscle samples, respectively; these values were lower than others from literature, where Cd concentrations reached 6 mg/kg, 4 mg/kg, and 1.4 mg/kg, for the liver, gills, and muscle, respectively (Arockia Vasanthi et al. 2013; Dural et al. 2006; Kalay and Canli 1999; Norouzi et al. 2017). Furthermore, no correlation with histological alterations was found in this study. Mercury content was very low and most of the samples fell under detection limits, especially for gills, and the maximum concentration reached was in liver samples from the Port of Genoa (0.28 mg/kg). Despite Hg mean levels in liver were lower than other studies on Mugilidae fish, where Hg content was recorded around 0.9 mg/kg (Durrieu et al. 2005; Mieiro et al. 2011), a positive correlation (*r*: 0.77, Spearman’s coefficient) was found between Hg content in liver and the presence of steatosis foci. An interesting situation was found in the present study regarding As mean concentrations, which were significantly higher, according to Mann–Whitney test, in individuals from the fishpond of S’Ena Arrubia than from the Port of Genoa, for both liver (*p* value: 0.00074) and gills (*p* value: 0.00028) (Table 2). No significant difference between sites was highlighted for muscle samples (*p* value: 0.54), where As content reached a maximum value of 3.21 mg/kg. Maximum concentrations of As found in all three tissues (7.34 mg/kg in liver and 3.65 mg/kg in gills from the fishpond; 3.21 mg/kg in muscle from the port), were higher in the present study than other findings from literature where As content never exceeded the values of 5 mg/kg, 1.5 mg/kg, and 2 mg/kg, for the liver, gills, and muscle, respectively (Engin 2015; Norouzi et al. 2017; Stancheva et al. 2013; Usero et al. 2003). Furthermore, As is known to be a non-essential metal which negatively affects the health status of organisms; however, no correlation was here found between As content and histological alterations. Another non-essential metal which is toxic for metabolism of organisms is Pb (Salem and Ayadi 2016) that here reached its higher concentrations in gills, as found in different studies (Bat et al. 2018; Engin 2015; Jelodar, et al. 2011); this may be due to the main absorption of Pb through water and its complexation with the mucus of the gill lamellae (Frías-Espericueta et. 2011). Lead concentrations were higher in samples from the port than from the fishpond, for the liver (*p* value: 5.608e − 13), gills (*p* value: 1.571e − 13), and muscle (*p* value: 3.667e − 09) (Table 2). In addition, Pb is assessed to alter normal histology of tissues (Authman et al. 2015; Gad El-Hak et al. 2021; Yacoub and Abdel Satar 2003), and, in the present study, a positive correlation of Pb content was found with the presence of necrosis foci in the liver (*r*: 0.55, Spearman’s coefficient) and SL epithelial lifting in gills (*r*: 0.50, Spearman’s coefficient). Furthermore, concentrations of Pb in samples from the port of Genoa were substantial when compared to other studies on Mugilidae fish, where a higher content of Pb is reported in gills, reaching a maximum of 3 mg/kg, while Pb concentrations in the liver and muscle usually do not exceed the values of 1 mg/kg and 0.3 mg/kg, respectively (Bat et al. 2018; Engin 2015; Licata et al. 2003; Rajeshkumar et al. 2015; Salem and Ayadi 2016; Stancheva et al. 2013; Usero et al. 2003). Another aspect that was investigated, concerning the concentration of metals found in the tissues, was the possible influence of size on the comparison between sites; in fact, since the fish from the port were bigger, and therefore presumably older, than those from the fishpond, they might show higher values of metals due to prolonged exposure (Wunderlich et al. 2015). This hypothesis was refuted by the statistical analysis that tested the correlation, using Kendall’s tau, between size (length/weight) of individuals and concentration of metals in different tissues. No correlation was found, except for a slight positive significant correlation between Pb gill content and both weight (tau: 0.37) and length (tau: 0.41). Nevertheless, linear regression showed a very low coefficient of determination for both weight (*R*^2^: 0.14) and length (*R*^2^: 0.22); therefore, the variance of Pb content in gills cannot be explained by size.

Although the Port of Genoa is closed to fishing, the S’Ena Arrubia fishpond is an area widely exploited commercially. Therefore, it was also investigated the presence of muscle samples that might exceed the legal limit provided for human consumption in terms of Cd, Hg, and Pb content. It was found that Cd and Hg content in all muscle samples was lower than the legal limit established for the edible part of fish, which is 0.05 mg/kg and 0.5 mg/kg respectively (European Commission 2008b). It was instead highlighted that three samples from the port but only one from the fishpond showed a Pb content in muscle which exceeded the legal limit of 0.3 mg/kg (European Commission 2008b). The present study provides a broad overview concerning the metal content in different tissues of specimens of the family Mugilidae, sampled at sites characterized by different levels of anthropization. This allowed a comparison between these sites and other similar studies in the literature and thus to investigate the exposure of organisms to these types of pollutants. Although studies that focus on the concentration of metals in water or sediments provide important data on the level of environmental contamination, only observing the presence of these contaminants within the tissues of organisms, it is possible to determine the actual exposure. In fact, the ability of metals to be transported within tissues, and thus influence the health of organisms, depends not only on their presence in the external environment, but even more on their form and the physiology of different species (Adams et al. 2010). Since biological responses are species-specific, however, it is difficult to determine tissue-based thresholds beyond which the content of a metal in a tissue can generate adverse health effects in the organism, as this differs greatly from species to species (Meador 2015), and knowledge in this regard is still limited.

### CYP450 induction and PAH metabolite analyses

**Fig. 1:**
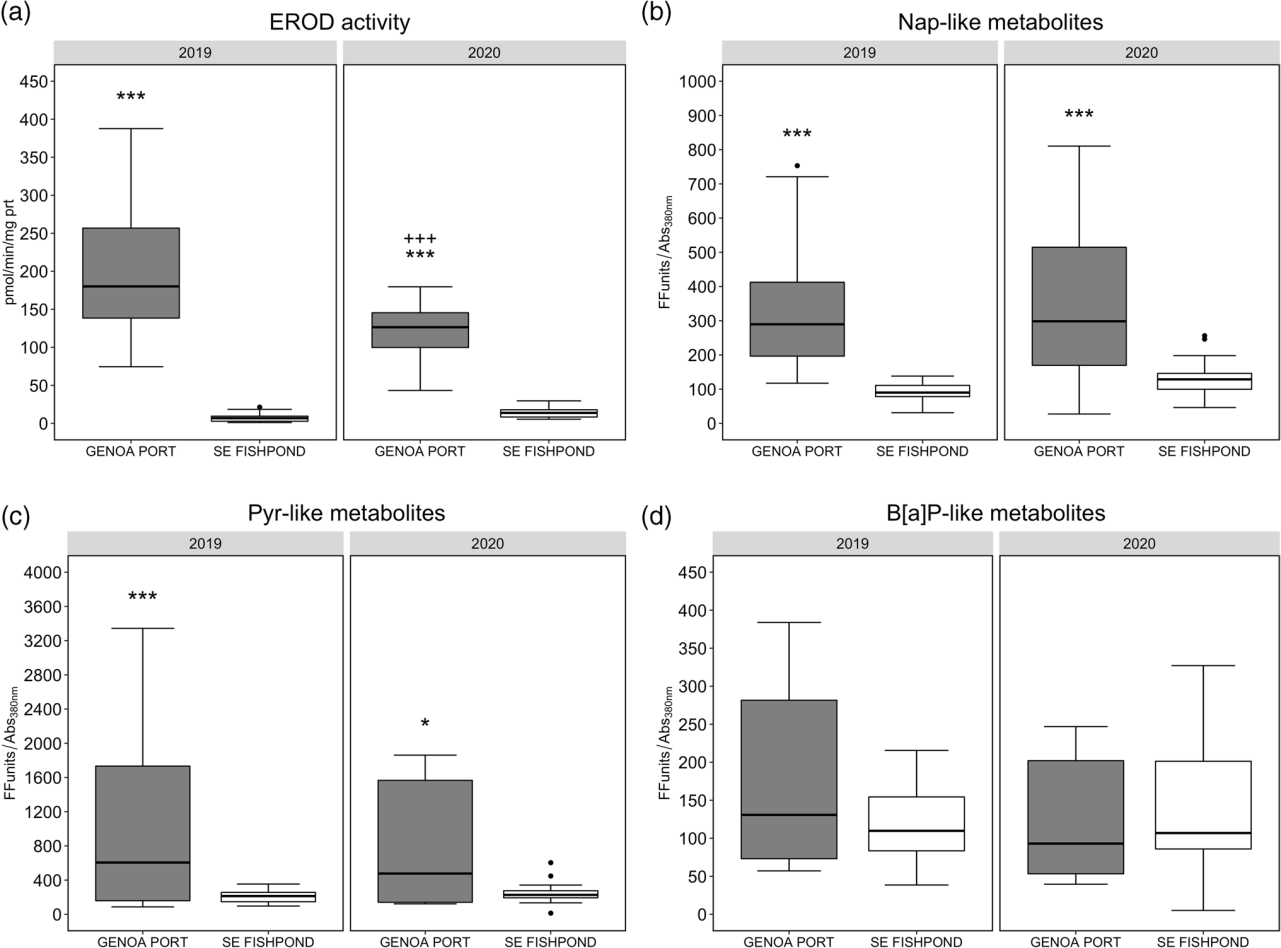
Comparison between site (GE: Port of Genoa port; OR: fishpond of S’Ena Arrubia) and campaigns for results from molecular analyses. **a** EROD activity in fish livers samples; **b** naphthalene-like metabolites in bile samples; **c** pyrene-like metabolites in bile samples; **d** benzo[a]pyrene-like metabolites in bile samples. Asterisks and crosses are used to highlight significant differences between sites (GE—OR) and years, respectively (* and + : *p* < 0.05; ** and + + : *p* < 0.01; *** and + + + : *p* < 0.001)

**Fig. 2:**
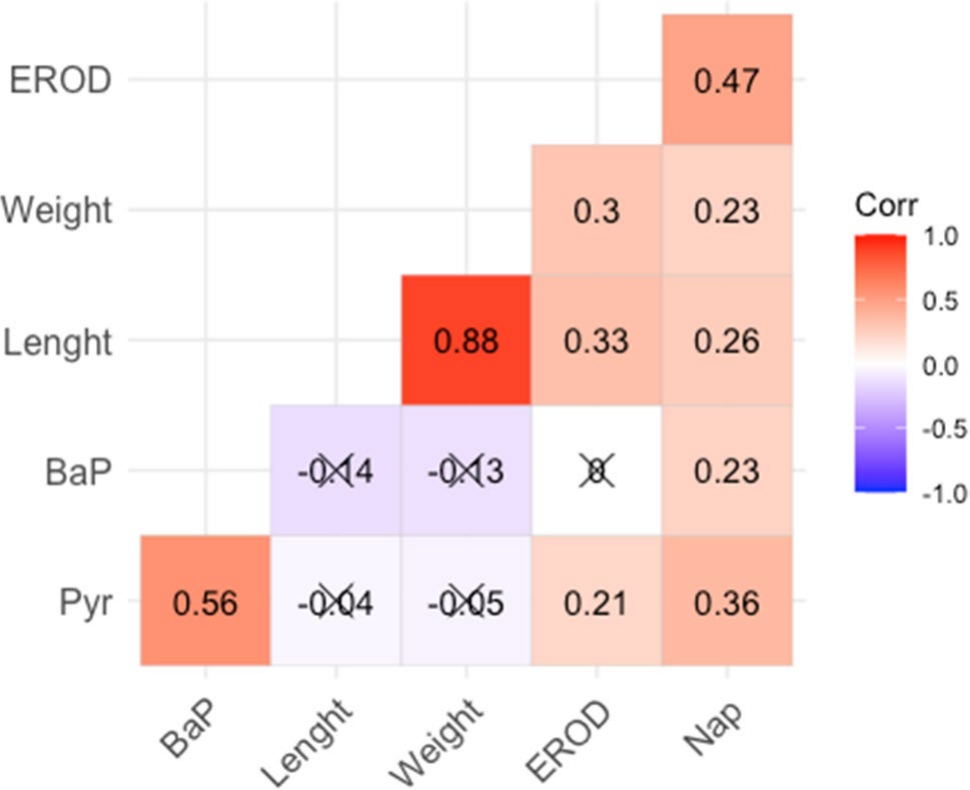
Kendall’s tau correlation heatmap showing correlation coefficients among biochemical parameters and individuals’ length and weight—blue and red indicate negative and positive correlation, respectively. Not significant correlations (*p* > 0.01) are highlighted by crossed correlation coefficients

A significant interaction between site and campaign of sampling was found for EROD activity (2-way ANOVA, *p* value = 0.000218): fish sampled in the Port of Genoa showed higher EROD activity compared to those sampled in S’Ena Arrubia fishpond in both the campaigns, and significant differences were found between 2019 and 2020 campaigns in Port of Genoa organisms (Fig. 1a). Mean values of EROD activity in the livers of Port of Genoa organisms were 193.47 ± 80.88 and 119.84 ± 35.28 pmol/min/mg prt, in 2019 and 2020, respectively, while a significantly lower EROD activity was measured in individuals from the fishpond of S’Ena Arrubia, with mean values of 7.18 ± 5.49 and 14.38 ± 7.21 pmol/min/mg prt in 2019 and 2020, respectively. Statistical analysis performed to test the comparison of EROD activity in males and females did not show a significant difference (Mann–Whitney test, *p* value: 0.06234). The EROD activity levels in Port of Genoa organism were generally higher compared to the baseline EROD activity observed in individuals of the same species collected from reference areas (Corsi et al. 2003; Gorbi et al. 2005; Pacheco et al. 2005; Yilmaz et al. 2016), and this suggests that the differences observed between sites were caused by exposure to organic compounds, as for instance petrogenic PAHs, known to promote the biotransformation mechanism of cytochrome P450A1 activity (Aas et al. 2000; Gorbi et al. 2005; Whyte et al. 2000). Indeed, EROD levels comparable to those measured in specimens sampled in the Port of Genoa were previously observed in mullets collected in polluted areas characterized by input of contaminants, as basins receiving sewage treatment plant discharge, industrial wastes, and urban outfall (Corsi et al. 2003; Yilmaz et al. 2016; Oliveira et al. 2020). There was no significant difference in EROD activity between species within the same site, for either the port (one-way ANOVA, *p* value: 0.6972) or the fishpond (one-way ANOVA, *p* value: 0.0689), confirming the validity of the comparison between sites. This hypothesis is reinforced by the higher degree of PAH metabolites in Port of Genoa organisms’ bile, which were always higher than those sampled in S’Ena Arrubia fishpond in terms of naphthalene- and pyrene-like metabolites (2-way ANOVA, *p* value = 2.77e − 09 and 0.000179, respectively) but not B[a]P-like ones (Fig. 1b-d). Mean naphthalene-like metabolite values (FF units/Abs_380nm_) in 2019 were 336.68 ± 190.81 and 89.82 ± 27.90, in respectively Port of Genoa and S’Ena Arrubia, while in 2020 were 342.41 ± 242.47 and 125.95 ± 49.08. Pyrene-like metabolites were found at higher values compared to naphthalene-like, but with a higher variability among samples, and mean values (FF units/Abs_380nm_) were 1045.54 ± 1067.92 and 755.55 ± 722.59 in Port of Genoa samples in 2019 and 2020, respectively, and 203.94 ± 73.70 and 247.90 ± 131.79 in S’Ena Arrubia organisms in 2019 and 2020, respectively. The presence of PAH metabolites in sampled specimens’ bile addresses thus the exposure to aromatic compounds and at the same time indicate a recent and ongoing contamination (de Albergaria-Barbosa et al. 2017; Johnson-Restrepo et al. 2008; Neves et al. 2007). In addition, Kendall’s Tau correlation highlighted a weakly positive significant relationship among individuals’ size (weight/length) and both EROD activity (tau: 0.30/0.33) and naphthalene-like metabolites (tau: 0.23/0.26) (Fig. 2). Nonetheless, the coefficient of determination of linear regression showed that length and weight do not explain the variability of EROD (*R*^2^: 0.21 and 0.09) and Nap-like metabolites (*R*^2^: 0.11 and 0.07). In addition, no significant correlation was found between weight/length and the other bile metabolites (Fig. 2). EROD activity was also found to be significantly correlated with Nap-like (Kendall’s tau: 0.47) and slightly with Pyr-like (Kendall’s tau: 0.21) metabolites (Fig. 2), linking CYP450 biotransformation pathway and PAH metabolites: beyond specific relationships between each analyzed metabolite and EROD activity, which may show asynchronous variations (Gagnon and Holdway 2000), these results suggest a high likelihood of PAHs being mainly responsible of EROD activity induction. Altogether, these results address a higher degree of organic contamination in Genoa port compared to S’Ena Arrubia fishpond, with very few differences between the two campaigns, confirming the suitability of EROD activity and PAH metabolites in monitoring activities of fish health status and environmental impacts. On the other hand, these results do not allow to exclude the presence and exposure of other organic contaminants typical of harbors that are known for their capability to induce EROD activity, as for instance planar PCBs (Gilroy et al. 2012; Mariottini et al. 2012).

### Histopathological analyses

**Fig. 3:**
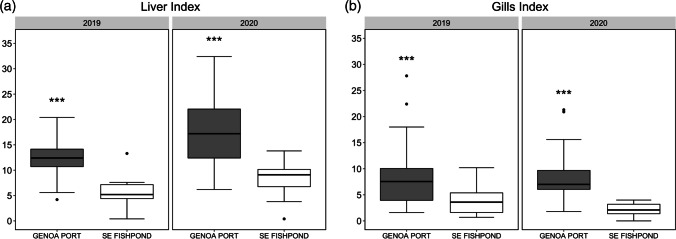
Comparison of histopathological indices between samples from the Port of Genoa and from the fishpond of S’Ena Arrubia, in 2019 and 2020 sampling campaigns. **a** Comparison of liver indices (*** *p* < 0.001); **b** comparison of gill indices (***p < 0.001)

Results from the histopathological analysis showed a worse health status of the liver and gills of fish from the Port of Genoa than those of the S’Ena Arrubia fishpond, in both 2019 and 2020 sampling campaigns (Fig. 3). These results were confirmed by the two-way ANOVA analysis which highlighted a strongly significant difference between the port area and the fishpond for both liver (*p* value 1.886e − 06) and gill (*p* value: 0.00061) indices, while no interaction resulted between sites and campaigns. Mean values of liver index for fish from the Port of Genoa were 12.4 ± 4.0 and 17.7 ± 7.3, in 2019 and 2020, respectively, while individuals from the fishpond of S’Ena Arrubia showed a liver index mean value of 5.4 ± 2.9 in 2019 and 8.3 ± 3.1 in 2020 (Table 1). Similarly, gill indices were lower for fish from the fishpond of S’Ena Arrubia, with mean values of 3.7 ± 2.6 in 2019 and 2.2 ± 1.2 in 2020, than for samples from the Port of Genoa which showed mean values of 8.7 ± 7.0 and 8.9 ± 5.7, in 2019 and 2020 campaigns, respectively (Table 1). Pearson’s correlation analysis between size of individuals and histopathological indices revealed a significant, although not particularly strong, positive correlation between length/weight and liver index (*r*: 0.48/0.44), and a weak correlation between length/weight and gill index (*r*: 0.30/0.20). Moreover, the coefficient of determination of linear regression showed that the variability of histopathological indices was not explained by length/weight, neither for liver (*R*^2^: 0.23/0.19) nor for gills (*R*^2^: 0.10/0.04). In addition, the one-way ANOVA test did not identify any significant difference between males and females concerning histopathological indices, either of the gills (*p* value: 0.168) or the liver (*p* value: 0.186). This result supports the fact that the difference identified, concerning the health status of the analyzed organs between sites, is indeed due to environment rather than age or physiology of individuals. Regarding liver histopathology, most of the samples from the Port of Genoa fell in the range that corresponded to slightly to moderately damaged tissue (10 < *I* ≤ 20), with 14 out of 19 samples in 2019 and 10 out of 18 samples in 2020, while few individuals presented moderate to heavily damaged liver tissue (20 < *I* ≤ 30), and only one sample had a liver index higher than 30 (2020 campaign). On the other side, almost all the liver samples from the fishpond showed an index lower than 10, which corresponded to normally or very mild altered tissue functionality, and only 1 out of 18 and 5 out of 18 samples, in 2019 and 2020, respectively, presented slightly to moderately damaged tissue (10 < *I* ≤ 20), with a maximum value of 13.8. In gill histopathological analysis, only one sample from the fishpond showed an index value higher than 10, and then, almost all fish were characterized by normally or very mild altered gill functionality; in contrast, 3 out of 20 and 2 out of 18 samples from the port, in 2019 and 2020 respectively, presented slightly to moderately damaged tissue (10 < *I* ≤ 20), and 4 samples (2 out of 20 in 2019 and 2 out of 18 in 2020) showed a gill index which corresponded to moderately to heavily damaged tissue (20 < *I* ≤ 30). Despite it is not possible to compare liver and gill indices, as they are based on different histological alterations, it is important to highlight that liver samples were overall more affected by some level of damage than gills. In fact, the main metabolic function of the liver is the detoxification of exogenous and endogenous substances, thus it is the main target for contaminants and consequently the most likely to undergo histological alterations, resulting in being the preferred organ to be studied to investigate the connection between environmental factors and metabolic dysfunction (Ardeshir et al. 2017; Saleh 1982).

**Fig. 4:**
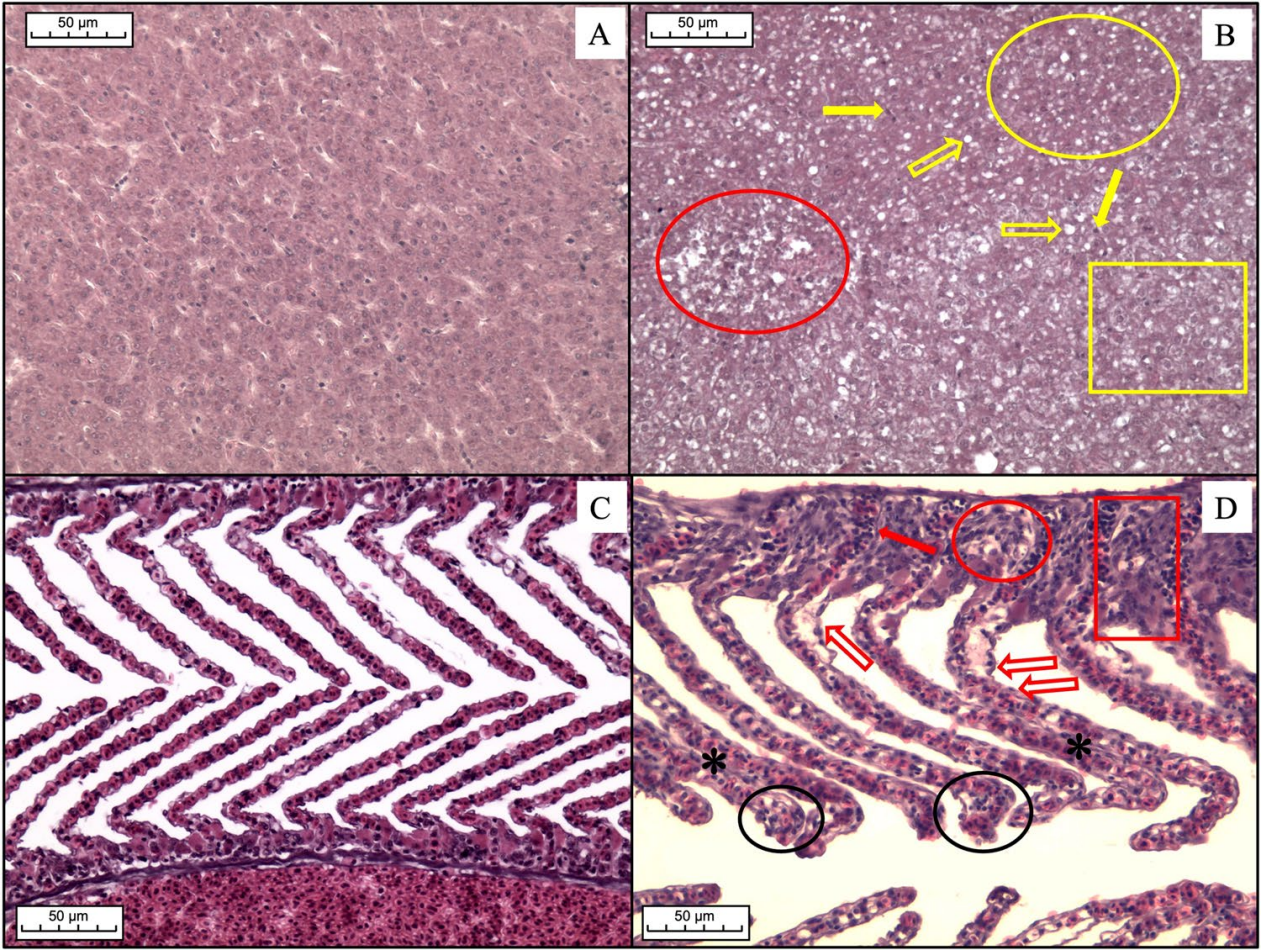
Examples of histopathology of liver and gills: **A** normal histology of liver from the fishpond of S’Ena Arrubia; **B** altered histology of liver from the Port of Genoa (yellow rectangle: loss of cord structure; yellow circle: degeneration; red circle: necrosis; yellow blank arrows: steatosis; yellow arrows: nuclei pyknosis); **C** normal histology of gills from the fishpond of S’Ena Arrubia; **D** altered histology of gills from the Port of Genoa (red rectangle: PL hyperplasia; black circles: SL hyperplasia; red circle: necrosis; red blank arrows: SL epithelial lifting; red arrow: granulocyte infiltration; asterisks: congestion)

**Fig. 5:**
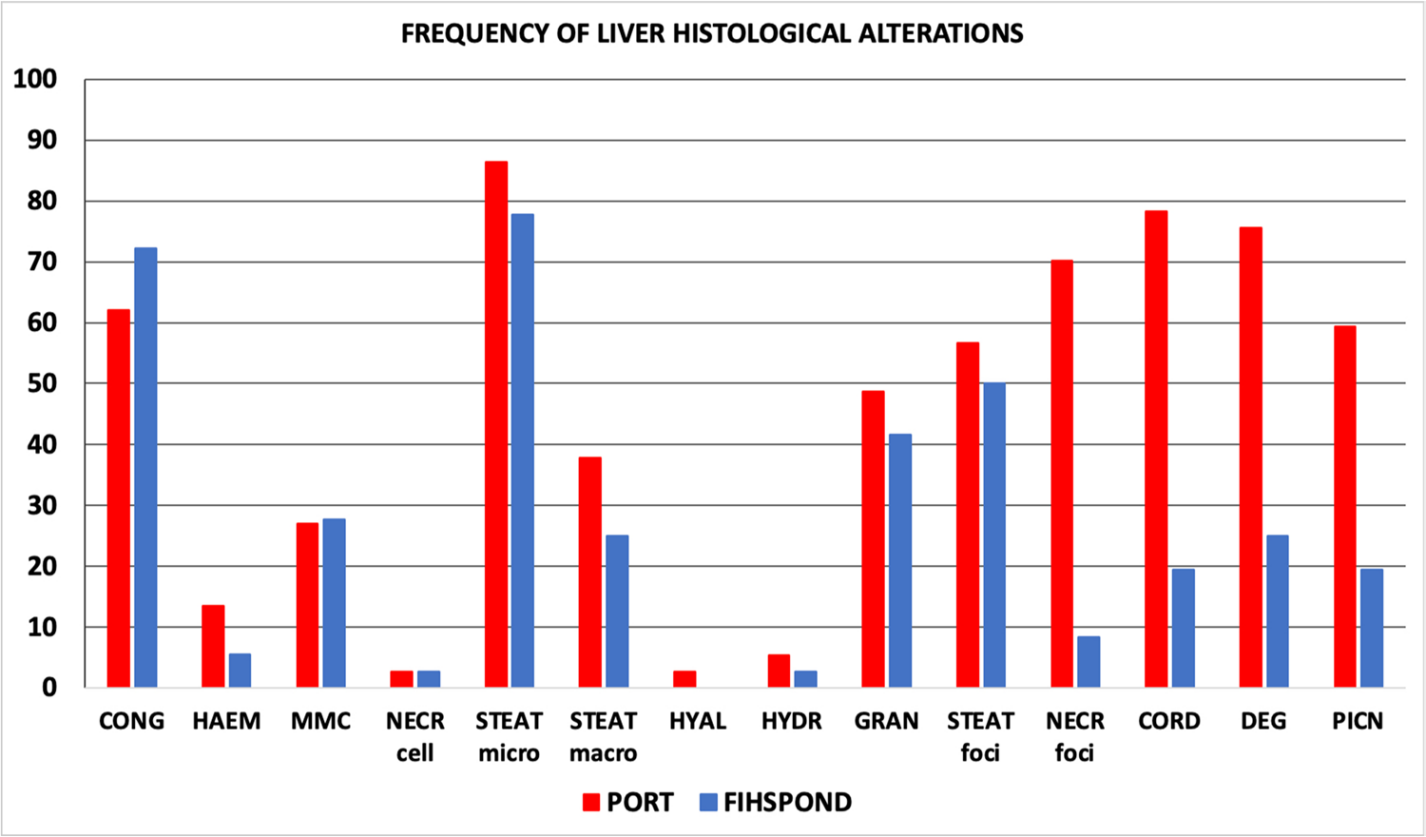
Histogram showing the frequency (%) of each liver histological alteration, in both sites (port and fishpond). Cong: vessels congestion; Haem: hemorrhage; MMC: centers of melano-macrophages; Necr cell: cellular necrosis; Steat: steatosis (micro, macro, or foci); Hyal: hyalinization; Hydr: hydropic change; Gran: granulocyte infiltrations; Necr foci: foci of necrosis; Cord: loss of cord structure; Deg: tissue degeneration; Picn: picnosis

**Fig. 6:**
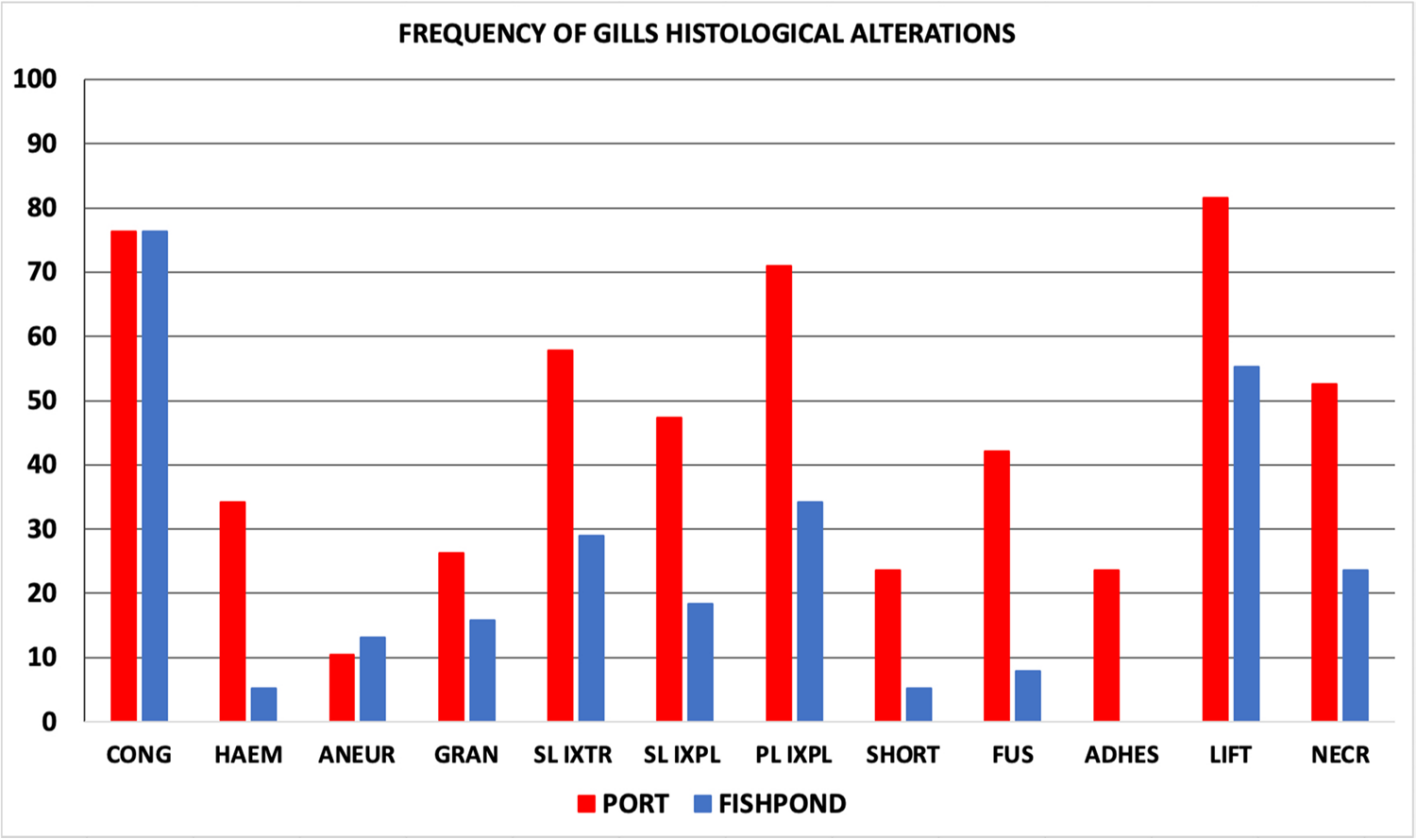
Histogram showing the frequency (%) of each gill histological alteration, in both sites (port and fishpond). Cong: vessels congestion; Haem: hemorrhage; Aneur: aneurisms; SL ixtr: secondary lamellae hypertrophy; SL ixpl: secondary lamellae hyperplasia; PL ixpl: primary lamellae hyperplasia; Short: shortening of secondary lamellae; Fus: fusion of secondary lamellae; Adhes: adhesion of secondary lamellae; Lift: lifting of secondary lamellae epithelium; Necr: necrosis

**Fig. 7:**
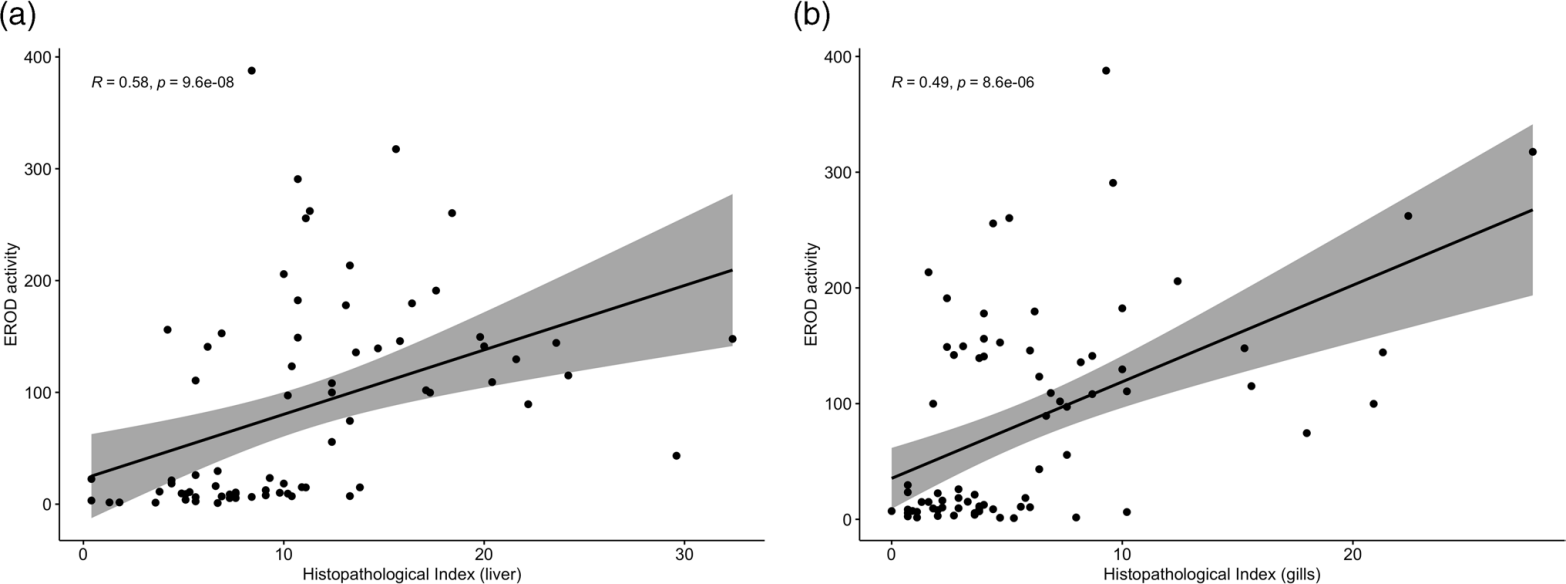
Results of Spearman correlation analysis between EROD activity and liver index (**a**) and EROD activity and gill index (**b**). Correlation coefficients (*R*) and *p* values (*p*) are given for each correlation

Liver histopathology revealed that samples from the Port of Genoa were more affected by histological alterations than samples from the fishpond of S’Ena Arrubia, as confirmed by PCA analysis. When differences between species were tested, *Chelon ramada* specimens appeared to have the most altered tissue, but this can be, at least partially, due to unequal representativity of individuals of each species which weakens the reliability of species comparison. In fact, when comparing liver indices between species within site, using one-way ANOVA, no significant difference was found for the port (*p* value: 0.3822), and difference for the fishpond was just slightly significant (*p* value: 0.0272). The same results concerning differences between sites and between species were shown by PCA analyses carried out on gill histopathology. Even for gill indices, interspecies variability within the same site is not significant, either for the port (*p* value: 0.839) or the fishpond (*p* value: 0.5297). Thus, the statistical analysis supports the validity of the comparison between sites. Main histological alterations in the liver of Mugilidae fish from the port were loss of the cord structure, nuclei pyknosis, granulocyte infiltration, congestion, tissue degeneration, steatosis, and necrosis (Figs. 4 and 5). Results are in accordance with findings from literature, as these types of histological changes in the liver are assessed to be a response of its metabolic function to the exposure of a polluted environment (Ben Ameur et al. 2012; Gad El-Hak et al. 2021; Hajirezaee et al. 2021; Rajeshkumar et al. 2015; Yacoub and Abdel Satar 2003). Lipid vacuolization was also found in several individuals from the fishpond of S’Ena Arrubia, but it was mainly present in the form of micro-steatosis, while macro-steatosis and foci of steatosis more frequently occurred in samples from the port (Fig. 5). This could be explained as lipid accumulation is a condition that normally occurs in liver tissue, due to its physiological role in the metabolism of endogenous molecules, but it happens to be increased by exposure to exogenous contaminants (Arockia Vasanthi et al. 2013). Few samples from the fishpond showed severe alterations, as degeneration of the tissue and necrosis, and with a smaller extent compared to the ones from the port. Gill histopathological analysis revealed that the most relevant alterations found in samples from the Port of Genoa were PL and SL hyperplasia, SL hypertrophy, SL epithelial lifting, and necrosis, followed by fusion and congestion of SL with occasional granulocyte infiltration (Figs. 4 and 6); results are in accordance with literature which highlighted the presence of the same alterations in fish exposed to a contaminated environment (Al-Ghanim et al. 2019; Ben Ameur et al. 2015; Gad El-Hak et al. 2021; Nagarjuna and Mohan 2017; Noguera et al. 2019). Hyperplasia and the consequent fusion of SL, SL hypertrophy, and SL epithelial lifting are defensive changes that occurs in gill tissue to increase the distance between blood circulation and the external environment (Arockia Vasanthi et al. 2013; Rajeshkumar et al. 2015). SL epithelial lifting frequently occurred with SL blood vessel congestion, as it is often the consequence of an edematous condition (Bhagwant and Elahee 2002). Despite gill alteration was more frequent in samples from the port, even most of the individuals from the fishpond showed the presence of congestion and SL epithelial lifting, but few fish were affected by severe modifications of gills (Fig. 6). Liver and gill histopathology effectively described the difference between a more polluted and a less polluted area, such as the Port of Genoa and the fishpond of S’Ena Arrubia. In fact, liver and gills are recommended for histopathological studies, since liver has a key role in detoxification metabolism and gills are the organ that is most exposed to the external environment (Arockia Vasanthi et al. 2013). A strong difference in the health status of fish organs between the two sites was highlighted, since the Port of Genoa is a highly anthropized area, where several industrial and commercial activities are present, as well as chemicals transported by natural rivers and sewage discharge, and pollutants coming from the city nearby; in contrast, the fishpond of S’Ena Arrubia arises in a natural environment, and no industrial activity directly affects the area. The Spearman analysis revealed a positive and significant correlation of the EROD activity with both liver (*R*: 0.58; *p* value: 9.6e − 08) and gill (*R*: 0.49; *p* value: 8.6e − 06) indices (Fig. 7). Increased EROD activity has been seen to correlate with damage and histopathological changes in literature. In the study of Au et al. (1999), the exposure of fish to B[a]P by intraperitoneal injection was followed by an enhanced enzymatic activity which showed a positive correlation with hepatocytological changes, as for example lipid vacuolization. The increased number of lipid droplets in liver tissue, together with capillary hyperemia, inflammatory response, cellular necrosis, and foci of cellular alteration, was also seen by Ortiz-Delgado et al. (2007) as related to EROD activity, following exposure to B[a]P-contaminated water. Other studies have analyzed fish exposure to field-collected sediments contaminated with various chemicals, such as polycyclic aromatic hydrocarbons (PAHs) and metals, revealing an induction of EROD activity correlated with the presence of histological changes in the liver and gills (Jiménez-Tenorio et al. 2008; Morales-Caselles et al. 2007). Evidence of a possible link between histopathological investigation and EROD analysis must however take into account that histopathology is a non-specific biomarker (Au 2004), and alterations of tissues were probably the consequence of exposure to a mix of contaminants and stressors, as would be expected in an environment as diverse as the Port of Genoa.

## Conclusions

The results obtained from this study carried out within the GEREMIA Project well represented the difference between the polluted area of the Port of Genoa, and a natural environment as the fishpond of S’Ena Arrubia. The integration of chemical analyses, together with CYP450 induction, biliary PAHs metabolite analyses, and histopathology, was confirmed as a reliable tool to investigate the health status of fundamental organs such as the liver and gills, strengthening the highlighted difference between sites and suggesting the presence of complex mixtures of contaminants that can affect organism functionality. Overall, the results obtained in this study allowed to validate this panel of analyses in a polluted context and propose its use as a tool for rapidly screening environmental quality, suggesting the reliability of biomonitoring using Mugilidae fish and multidisciplinary approaches. At the same time, when results obtained with this approach suggest possible negative effects on biota, these should be supported with further more in-depth investigations, aimed to precisely characterize the levels of different typologies of contaminants in abiotic and biotic matrices and define effects charging multiple biological traits on a battery of model species that should be the most diverse possible. In conclusion, the panel of analyses exploited in the present study was assessed to be a rapid, suitable, and useful tool for monitoring of anthropized environments using Mugilidae fish as bioindicator, able to highlight the presence of different typologies of contaminants and the onset of biological effects through different mechanisms, that could be widened to other typologies of investigations specific for each case study.

## Data Availability

The authors confirm that the data supporting the findings of this study are available within the article supplementary materials and on request from the corresponding author, A.R.

## Declarations

### Data transparency

All authors agreed to be accountable for all aspects of the work in ensuring that questions related to the accuracy or integrity of any part of the work were appropriately investigated and resolved.
